# Immunomodulatory effects of inactivated *Ligilactobacillus salivarius* CECT 9609 on respiratory epithelial cells

**DOI:** 10.1186/s13567-023-01228-z

**Published:** 2023-10-16

**Authors:** María Bravo, Selene Diaz-Chamorro, Sergio Garrido-Jiménez, Javier Blanco, Irene Simón, Waldo García, María José Montero, Pilar Gonçalves, Carlos Martínez, Guadalupe Cumplido-Laso, Dixan Agustín Benítez, Sonia Mulero-Navarro, Francisco Centeno, Ángel Carlos Román, Pedro Fernández-Llario, Rosario Cerrato, José María Carvajal-González

**Affiliations:** 1 Departamento de Bioquímica, Biología Molecular y Genética, Facultad de Ciencias, Universidad de Extremadura, 06071 Badajoz, Spain; 2 Ingulados S.L., Cáceres, Spain

**Keywords:** Airway epithelial cells, multiciliated cells, secretory cells, immunomodulator, lactic acid bacteria, *Pasteurella multocida*

## Abstract

The microbiota in humans and animals play crucial roles in defense against pathogens and offer a promising natural source for immunomodulatory products. However, the development of physiologically relevant model systems and protocols for testing such products remains challenging. In this study, we present an experimental condition where various natural products derived from the registered lactic acid bacteria *Ligilactobacillus salivarius* CECT 9609, known for their immunomodulatory activity, were tested. These products included live and inactivated bacteria, as well as fermentation products at different concentrations and culture times. Using our established model system, we observed no morphological changes in the airway epithelium upon exposure to *Pasteurella multocida*, a common respiratory pathogen. However, early molecular changes associated with the innate immune response were detected through transcript analysis. By employing diverse methodologies ranging from microscopy to next-generation sequencing (NGS), we characterized the interaction of these natural products with the airway epithelium and their potential beneficial effects in the presence of *P. multocida* infection. In particular, our discovery highlights that among all *Ligilactobacillus salivarius* CECT 9609 products tested, only inactivated cells preserve the conformation and morphology of respiratory epithelial cells, while also reversing or altering the natural immune responses triggered by *Pasteurella multocida*. These findings lay the groundwork for further exploration into the protective role of these bacteria and their derivatives.

**Supplementary Information:**

The online version contains supplementary material available at 10.1186/s13567-023-01228-z.

## Introduction

The airway tract and its epithelia, the airway epithelium, constitute the frontline in the interaction between living organisms and the environment [1]. The airway epithelium represents the first cellular barrier, but also one of the first molecular responders [2–4]. In this pseudostratified epithelium, two major cell types put in place a mix of a mechanical and molecular response. Secretory cells secrete mucus to trap pathogens and exogenous particles, including pollutants and dust. Furthermore, multiciliated cells (MCCs), by using their apical ciliary projections, propel this mucus out of the organism [5–7]. In those events where this mechanism is not sufficient and the infection occurs, bacterial or viral infection, an immune reaction is triggered within the epithelium. Those early events initiate the so called innate immune response [2, 3].

The innate immune response in the airway epithelium aims to clearing the inhaled pathogens and harmful pollutants to maintain homeostasis in the lung [8, 9]. The airway epithelium has receptors and antimicrobial compounds which constitute the innate immune system. Numerous innate immune receptors such as the Toll-like receptors (TLRs) and nucleotide oligomerization domain–like receptors (NLRs) are expressed by the airway epithelium. These receptors induce the production of proinflammatory cytokines and chemokines (e.g., IL-6, CXCL8, IL-1b, GM-CSF, and G-CSF), affecting directly microorganisms and recruiting immune cells, such as neutrophils and T cells [2]. Proinflammatory/TLR signalling also regulates mucin gene expression. Therefore, MUC5AC and MUC5B are produced by secretory epithelial cells lining the airways and submucosal glands, contributing to the mucus layer [10]. Moreover, the apicolateral border of airway epithelial cells has tight and adherence junctional complexes, which are important to prevent paracellular invasion of inhaled pathogens [8].

*Pasteurella* species are part of the normal microbiota of the oral, nasopharyngeal and upper respiratory tract of many wild and domestic animals [11]. Many *Pasteurella* species are opportunistic and can cause a wide range of diseases (pasteurellosis). *Pasteurella multocida* (*P. multocida*), a gram-negative coccobacillus, is a common respiratory pathogen in animals causing pneumonia [12]. *P. multocida* zoonotic threat comes from animal bite, scratch wounds or respiratory exposure, but does not represent a common respiratory pathogen. Instead, it can be considered as a potentially severe coinfection agent with either viral or bacterial infections [13]. Out of the five serogroups described (A–F), serogroup A and D are the most associated to human and animal infections [11, 12]. Although the bacterium has been known for decades, the pathogenesis and the molecular mechanisms of *P. multocida* induced host immunity are poorly understood.

Although several microorganisms from microbiota are partly responsible for the development of multiple physiological processes within their host, only certain bacterial groups, such as lactic acid bacteria (LAB), interact with the mucosal immune system. Host-microbiota crosstalk at mucosa surfaces has been widely studied on intestinal epithelium, but little is known about the interaction between LAB and respiratory epithelial cells. In our previous work, we provided a very comprehensive phenotypic and genotypic characterization of the immunomodulatory properties of a selection of LAB isolates. A strain of *Ligilactobacillus salivarius*- later registered at Spanish Type Culture Collection CECT 9609- showed interaction with phagocytes, including macrophages. Also, among all isolates, this strain displayed the greatest extracellular antipasteurella activity measured by broth microdilution test [14].

Here, we search for new natural products obtained from *L. salivarius* CECT 9609 to combat this infection at the molecular level with minimal negative effects on the biology/physiology of the airway epithelia. In addition, we characterize at the cellular and molecular level the infection mediated by *Pasteurella multocida* in airway epithelia at early stages.

## Materials and methods

### Mouse tracheal epithelial cell (MTECs) primary cell preparation

The treatment of animals used in these studies follows the National and European legislation (Spanish Royal Decree RD53/2013 and EU Directive 86/609/CEE as modified by 2003/65/CE, respectively) and the Institute of Laboratory Animal Resources (ILAR) for the protection of animals used for research. Furthermore, all applicable international, national, and/or institutional guidelines for the care and use of animals were followed. The experimental protocols applied in this work were approved by the Bioethics Committee for Animal Experimentation of the University of Extremadura (Registry July 7^th^, 2017). Adults wild-type C57BL/6J mice provided MTECs. Firstly, tracheas were dissected from the larynx to the bronchial main and, subsequently, collected in cold Ham-F12 1X (Gibco, ThermoFisher Scientific, Waltham, MA, USA) and supplemented with penicillin and streptomycin 1% (P/S, Gibco). Additionally, cold Ham-F12 P/S was used to removed connective, fatty and vascular tissues.

The next step was to cut longitudinally the clean tracheas which were incubated in Ham-F12 P/S containing 1.5 mg/mL pronase (Roche Molecular Biochemicals, Basel, Switzerland) for 16 h at 4 °C. After this time, 10% Foetal Serum Bovine (FBS, Gibco) was added to Ham-F12 P/S to stop the pronase activity. Then, supernatant was transferred to a new sterile tube and, in addition, Ham-F12 with 10% FBS was added to the tracheas with the goal to obtain more cells. After that, supernatant of both tubes was mixed and centrifuged at 500 × *g* for 5 min. The following step was to resuspend the pellet containing cells in 5 mL of Ham-F12 P/S with 0.5 mg/mL pancreatic DNAse I (Sigma, St. Louis, MO, USA), incubated for 10 min room temperature and centrifuged at 500 × *g* for 5 min. After this time, cells were resuspended in Pneumacult-Ex Plus complete medium (StemCell, Vancouver, Canada) and, to remove fibroblasts, they were preselected in Primaria tissue culture plates (Corning, New York, NY, USA) for 4 h in 5% CO_2_ at 37 °C. Finally, the non-adhered cells were transferred in 60 mm plates previously treated with 50 µg/mL type I rat tail collagen (Gibco) in 0.02 N acetic acid.

### Cell culture, differentiation and treatments

MTECs were cultured and expanded in Pneumacult-Ex Plus medium at 37 °C in 5% CO_2_. This medium was replaced every 2 days until 70–80% confluence. To provide separation of cell- cell junctions, cells were incubated with 0.02% EDTA in PBS for 20 min at 37 °C. Then, PBS-EDTA was removed and, subsequently, accutase (Gibco) was added and incubated for 10 min at room temperature to separate cells from plate. After that, suspension cells were centrifuged at 1000 × *g* for 5 min and pellet containing cells was resuspended in 1 mL of Pneumalcult-Ex plus and counted. Cells were seeded at a confluence of 9 × 10^4^ cells/cm^2^ in polyester porous membranes (Transwell 0.4um pores, Corning). Every two days, upper and lower chambers were filled with Pneumacult-Ex Plus medium. When porous membranes were at confluence (4–6 days), medium was removed from the upper chamber to simulate the organization of respiratory tract tissue. For differentiation, ALI medium (Air–Liquid Interface Medium, StemCell) was added to the lower chamber, which was replaced every two days until the end of the differentiation for 14 days (ALI 14).

### *Pasteurella multocida* infection/exposure to epithelial cells

*Pasteurella multocida* was isolated from a clinical outbreak of pasteurellosis in a lamb feedlot causing high morbidity rates. *P. multocida* serotype A was confirmed by PCR [15] and cryo-stocked at −80 °C. Cryo-preserved stocks were recovered in Columbia agar supplemented with 7% sheep blood (Oxoid, ThermoFisher Scientific, Waltham, MA, USA) at 37 °C for 24 h. A pure colony was then inoculated to Brain Heart Infusion (BHI, Scharlab, Sentmenat, Barcelona, Spain) and incubated at 37 °C for 48 h. Medium was discarded after centrifugation at 5000 rpm for 10 min and pellets were adjusted to serial concentrations.

### LS culture and subproducts and treatments

The LS isolate is the registered lactic acid bacteria *Ligilactobacillus salivarius* CECT 9609. In a previous study, LS displayed in vitro inhibitory effect against *P. multocida* and interaction with innate immune system cells and pathways [14]. Three subproducts from LS were obtained: cell-free supernatants (SN) and microbial cell pellets, either alive (A-LS) or inactivated (i-LS). LS was inoculated onto De Man, Rogosa and Sharpe agar medium (MRS, Scharlau, Scharlab) at 37 °C for 24 to 48 h. A pure colony was propagated in MRS broth and incubated at 37 °C for 72 h with aeration. Cell-free SN was collected from 72-h MRS cultures after centrifugation at 5000 rpm for 10 min and filtration through 0.22-μm-pore-size syringe filters (Branchia, Labbox, Premia De Dalt, Barcelona, Spain). Microbial cell pellets were also collected for A-LS product. For i-LS product, 72-h MRS cultures were heat-inactivated at 80 °C for 2 h and microbial cells were collected after centrifugation at 5000 rpm for 10 min.

### Immunofluorescence of airway epithelial monolayers

Air–liquid interface (ALI) cultures of MTECs were firstly washed using PBS Ca^+2^/Mg^+2^ to preserve cell–cell junctions. After that, the cells were fixed in 4% paraformaldehyde (PFA, Polyscience, Niles, IL, USA) for 15 min at room temperature, permeabilized in PBS-Triton 0.1% for 15 min and blocked in PBS-Triton with 2% bovine serum albumin (BSA, Roche) for 1 h. Then, polyester porous membranes on which the cells grew were separated of the supports with a scalpel and placed in a wet chamber in dark. Samples were blocked in PBS-Triton with 2% bovine serum albumin (BSA, Roche) for 1 h and, subsequently, they were incubated with primary antibodies anti-Acetylated-Tubulin (1:100) diluted in PBS-Triton-2%-BSA overnight at 4 °C. After this time, samples were washed five times in PBS-Triton and incubated with fluorescent secondary antibodies Alexa-Fluor 488 anti-Mouse [Invitrogen, ThermoFisher Scientific, Waltham, MA, USA (#A11001, 1:500)] diluted in PBS-Triton-2%-BSA. In this same solution, Fluor 594 Phalloidin (Invitrogen, #A12381, 1:500) was utilized to label actin cytoskeleton, and 0.5 µg/mL 4′,6-diamidino-2-phenylindole (DAPI, Thermo Scientific, #62,248, 1:2000) to label nuclei. After 1 h of incubation at room temperature, samples were washed five times in PBS-Triton again and mounted in Vectashield (Vector Labs, Newark, CA, USA). Finally, an Olympus FV 1000 confocal microscope was used to take images, which were processed using ImageJ (Fiji, Bethesda, MD, USA) and Adobe Photoshop CC 2019.

A MATLAB function was designed to quantify the number of cells per images throughout the cell proliferation process. For that, this function can automatically pre-process DAPI-stained multiple images and normalize them by an average nuclei size. This MATLAB function is available on request at Ingulados webpage.

### Scanning electron microscopy (SEM) of airway epithelial monolayers

Differentiated MTECs at ALI 14 were washed with PBS Ca^+2^/Mg^+2^, keeping them inside transwells plate. Then, cells were fixed in 2.5% glutaraldehyde for 90 min at 4 °C in a solution of 0.2 M cacodylate. After that, samples were washed five times for 5 min using 0.2 M cacodylate and stained with 1% osmium tetroxide (Sigma) in 0.2 M cacodylate for 2 h at 4 °C. Then, they were dehydrated with increasing concentrations ethanol, from 10 to 100%, for 30 min at 4 °C and dried by liquid carbon dioxide critical point. Finally, samples were gold sputter coated, and visualized in a Quanta 3D FEG (ESEM-FIB; FEI Company, Hillsboro, OR, USA).

### TEER measurements of airway epithelial monolayers

Transepithelial electrical resistance (TEER) was used to infer the integrity of tight junction permeability along differentiation process of MTECs. TEER values in Kilohms (kΩ) were measured every two days until ALI14. The first step was to wash once the upper chamber of transwells in PBS before starting the measurements. Then, the PneumaCult medium was replaced by DMEM medium at 37 °C, which was added to the upper and lower chamber of transwells. At this point, TEER was measured with Evom3, positioning electrode in the upper chamber. For each record, a fold change relative to control cells was determined. Finally, the results were shown by graphs using Microsoft Excel (Redmond, WA, USA) and GraphPad Prism 8 (Boston, MA, USA).

### Transcriptional analysis

Ilustra RNAspin Mini Kit (GE Healthcare, Chicago, IL, USA) was used to isolate total RNA from MTECs. After elution of total RNA in 40 µL of RNase-free water, 200–400 ng RNA was transcribed into cDNA using High-Capacity cDNA reverse transcription (Applied Biosystems) following the manufacturer’s instructions. Quantitative PCR (qPCR) was utilized to analyse gene expression by means of PowerUp SYBR Green PCR Master Mix (Applied Biosystems, ThermoFisher Scientific, Waltham, MA, USA) in a QuantStudio3 (ThermoFisher) with specific primers. The setup of PCR reaction consisted of 50 °C for 2 min, 95 °C for 10 min, and 50 cycles of 95 °C for 15 s and 60 °C for 1 min. The specificity of reaction was confirmed by Melt curve analysis.

RNA-seq was performed using Illumina technology (10 M reads per sample, 2 × 75 bp) according to Novogene Inc. (Beijing, China) recommendations. Mouse mm10 genome and Kallisto pseudo-alignment software [16] were used to process the raw FASTQ reads by direct quantification in RPKM per transcript. Then, differentially expressed genes (DEGs) were inferred using a negative binomial distribution [17] with adjusted *p*-value below 0.05 and absolute value of log_2_ of fold change between conditions of at least 1. Subsequently, DAVID [18, 19] and STRING [20, 21] were employed to analyse the obtained DEGs by their Gene Ontology. Finally, differences between the samples used for RNA-seq were visualized by tSNE. Datasets generated during the current study are available from the corresponding author on reasonable request.

### Statistical analyses

Different experimental groups (GraphPad Prism) were compared against control conditions by means of two-tailed *t*-tests. qPCR experiments were made with at least 4 biological replicates while each qPCR reaction was performed with 2–3 technical replicates. Relative expression was obtained using EIF1a as a housekeeping gene.

## Results

### LS, alive or inactivated, does not affect airway basal stem cell (BSCs) proliferation

In early 2018, Ingulados Research S.L. isolated a collection of bacteria with potential benefit to treat livestock and wildlife animals. Of that collection *L. salivarius* CECT 9609, named LS, showed potential benefits to treat/prevent certain common infections. Based on this preliminary data, we decided to closely analyse the interaction with the airway epithelium, which is a prime target for common infections. For this matter we decided to use three different products out of LS cultures. Those three products were alive bacteria (A-LS), fermentation products (bacteria supernatants, SN) and heat-inactivated bacteria (i-LS).

**Figure 1:**
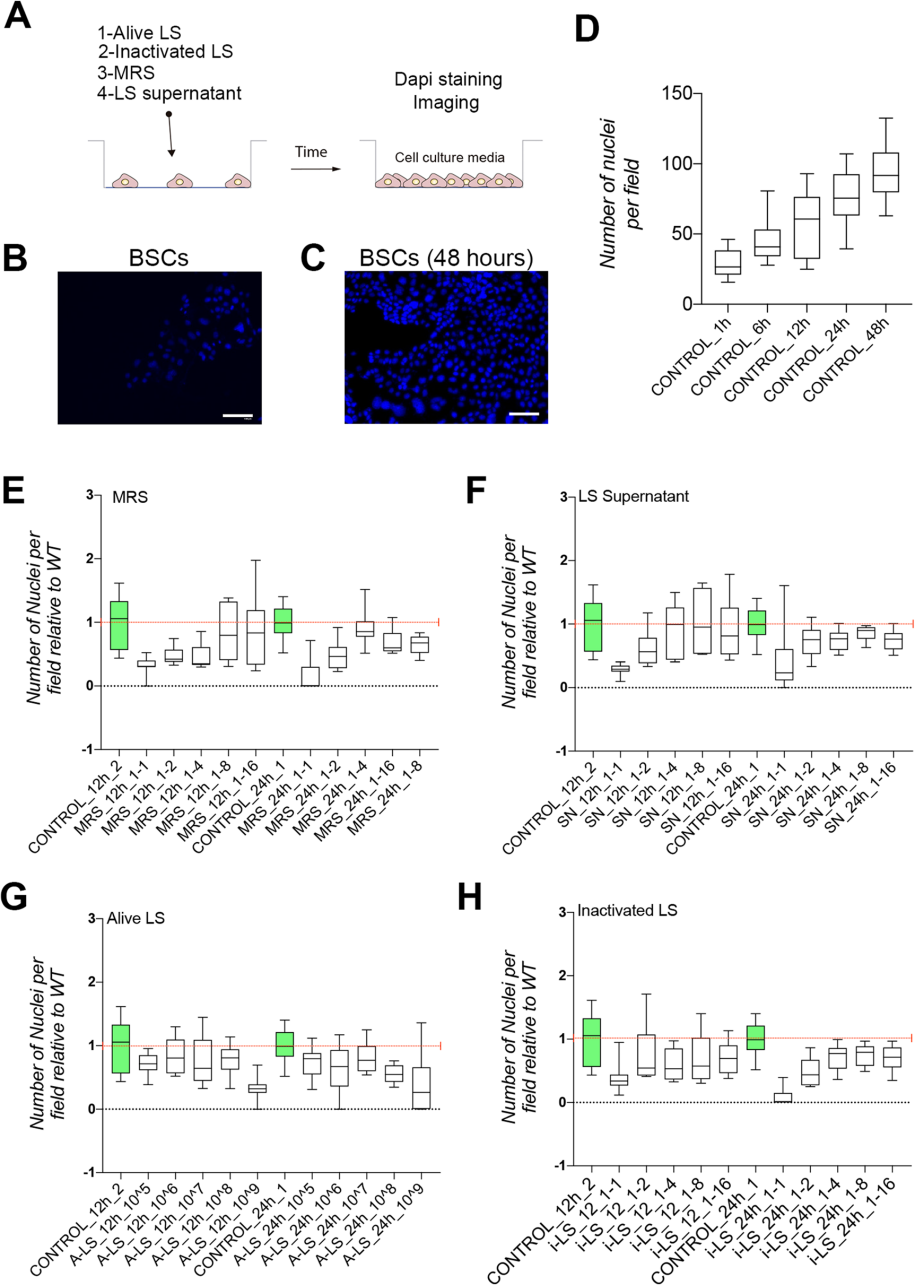
**LS or inactivated LS do not affect airway basal stem cell (BSCs) proliferation. A** Schematic representation of the basal stem cells (BSCs) treatment and processing protocol. **B**, **C** Images of DAPI labelled cells in control treatment for 1 h (**B**) and 48 h (**C**). **D** Quantification of cell numbers in control conditions at different time points using our semi-automatic system (see materials and methods for more detail). **E–H** Cell number quantification at two different times and relative to control conditions in MRS medium (**E**), LS supernatant (**F**), alive LS (**G**) and inactivated LS (**H**). Scale bar in B and C represents 100 μm.

To establish the potential cytotoxic effect of those different products, we started by assessing their impact on basal stem cell proliferation. Basal stem cells are responsible for airway epithelia regeneration producing secretory cells and ciliated cells through a cell differentiation process [22, 23]. We used primary airway basal epithelial cells to measure cell proliferation under different culture conditions. To be able to test multiple conditions, we developed a script to automatically count cell number using fluorescently labelled cells and imaged them in a EVOS XL FLOID microscope. First, we assessed cell proliferation in control conditions to demonstrate that we could monitor cell culture growth (Figures 1B, C). Next, we tested more than 100 experimental conditions using different products, dilutions, and bacterial density. Based on our image analyses, we found that the MRS medium alone, the medium where LS is grown, significantly decreased BSCs proliferation 12 h after exposure (Figure 1E). Similar results were obtained with LS fermentation products (supernatant, SN), although we found that this medium, which was modified by LS, is less aggressive than the MRS alone (Figure 1F).

Next, we tested both alive and inactivated LS in our BSCs proliferation model system. We found that treatment with alive LS (A-LS) retained cell proliferation (above 80% compared to WT) in close to control conditions up to 10^8^ A-LS for 12 h and 10^7^ A-LS for 24 h (Figure 1G). Finally, we tested inactivated LS (i-LS) and found no major defect on cell proliferation after 24 h of treatment with a proliferation above 80% relative to control conditions (Figure 1H).

Taking into consideration all the proliferation results, we decided to continue with A-LS and i-LS products and discard the fermentation products.

### Alive LS treatment diminishes the epithelial barrier function of the airway epithelia

**Figure 2:**
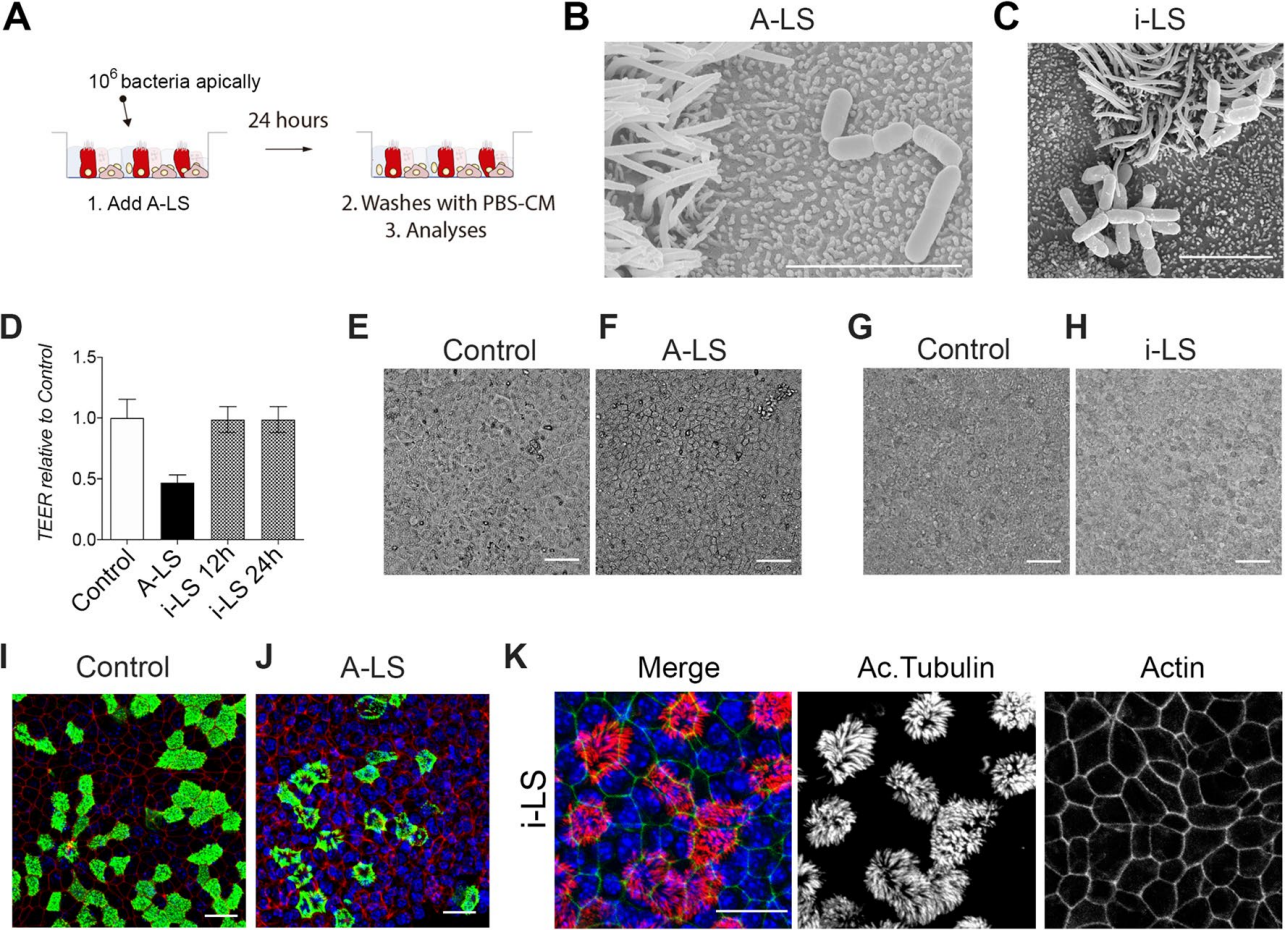
**A-LS cells affect epithelial barrier function and multiciliated cells. A** Schematic representation of the airway epithelial monolayers treatment and processing protocol with alive (A-LS) and inactivated (i-LS) bacteria. **B**, **C** Scanning electron microscopy images of airway epithelial cells treated with A-LS (**B**) or i-LS (**C**). **D** Transepithelial resistance (TEER) measurements were used to test the epithelial barrier function in airway control epithelial cells or treated with A-LS or i-LS. **E–H** Images of live cells in control conditions (**E** and **G**), treated with A-LS (**F**) or treated with i-LS (**H**). **I**, **J** Maximal projection of confocal images for acetylated tubulin (in green), phalloidin (in red) and nucleus (in blue) in controls (**I**) and A-LS treated cells (**J**). **K** Maximal projection of confocal images for acetylated tubulin (in red), phalloidin (in green) and nucleus (in blue) in i-LS treated cells. Scale bar in B and C represents 5 μm. Scale bar in E, F, G and H represents 50 μm. Scale bar in I, J, and K represents 20 μm.

Once we had determined the effect on BSC proliferation, we evaluated the effect on the airway epithelial barrier function by adding A-LS or i-LS at the apical side of the epithelia (Figures 2A–C). To achieve this, we measured the transepithelial resistance in control versus treatment, A-LS (alive LS) and i-LS (inactivated LS). We found that treatment for 12 h with A-LS reduced the TEER values by a half (Figure 2D). However, i-LS does not affect the barrier function (no significant changes in TEER values) after 24 and 48 h of treatment (Figure 2D). Direct observation under the microscope showed morphological changes in the airway epithelium that were not observed after i-LS treatment (Figures 2G, H). However, the intercellular space was more evident in A-LS treated epithelia (Figures 2E, F), which is consistent with a decrease in TEER.

To further characterize and confirm those morphological changes induced by A-LS treatment, we processed the epithelial monolayer for confocal microscopy to image the actin cytoskeleton, cilia distribution and morphology and the nuclei. We found that A-LS altered the actin cytoskeleton of the monolayer and the cilia pattern in MCC (Figure 2, panel J and Additional file 1) when compared to control monolayers. Conversely, when we performed the same analyses in i-LS treated cells, we did not find any actin cytoskeleton and cilia related phenotypes, when compared to control conditions (Figure 2K). The A-LS effect on the cilia distribution at the apical membrane of MCC was confirmed using scanning electron microscopy imaging (Additional file 1).

Based on our results, we found that A-LS treatment significantly impairs the airway epithelial barrier function, as evidenced by a substantial decrease in transepithelial resistance (TEER) values and observed morphological changes. In contrast, inactivated i-LS treatment does not affect the barrier function or induce similar morphological alterations.

### *Pasteurella multocida* infection characterization in airway epithelial cells

**Figure 3:**
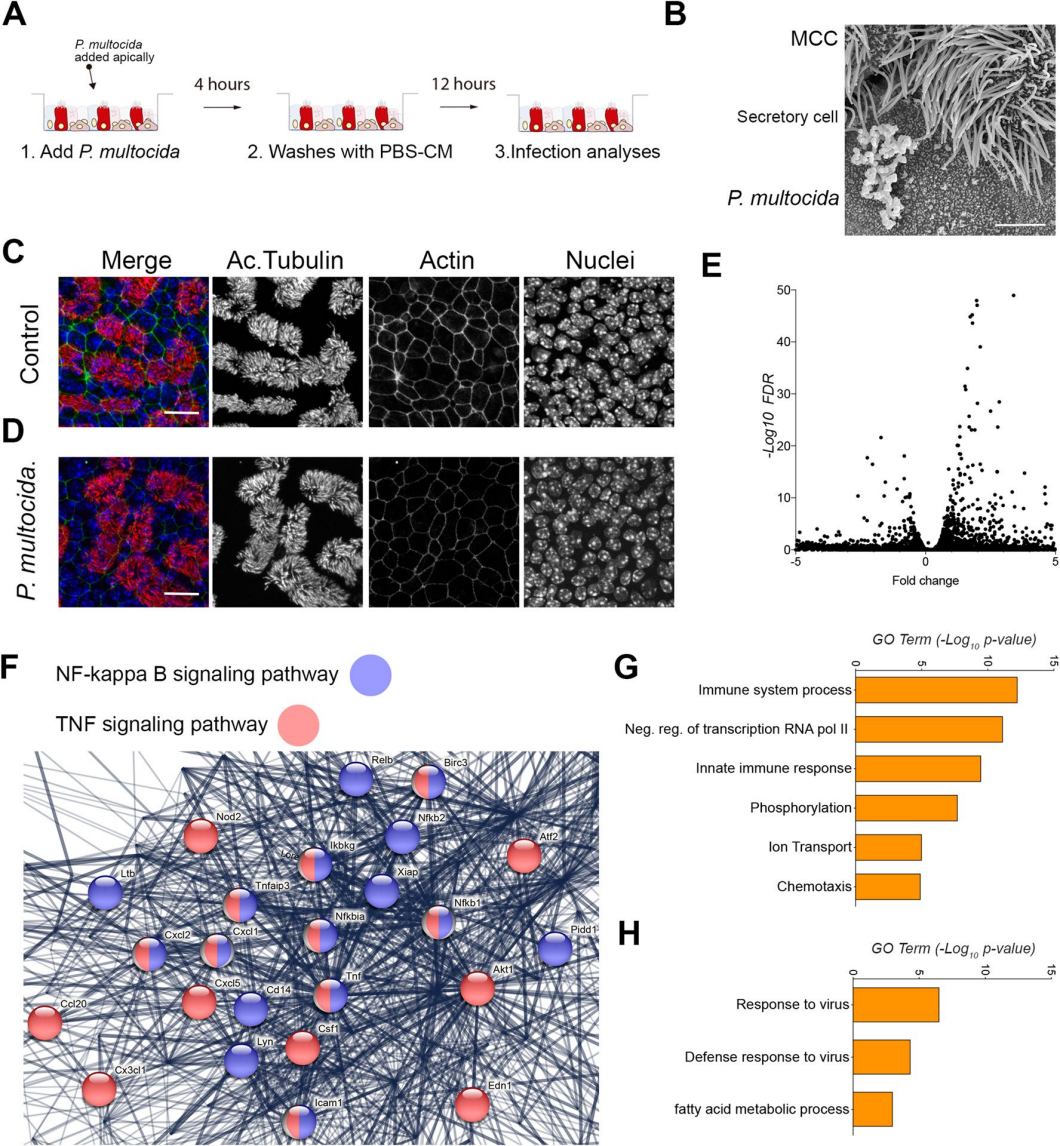
***Pasteurella multocida infection characterization in airway epithelial cells. A*** Schematic representation of the *Pasteurella multocida* infection protocol. **B** Scanning electron microscopy image of mouse tracheal epithelial cells infected with *P. multocida* (**C**, **D**) Maximal projection of confocal images for acetylated tubulin (in red), phalloidin (in green) and nucleus (in blue) in controls (**C**) and *P. multocida* infected (**D**) MTECs. **E** Volcano plot of control vs *P. multocida* infected MTECs. **F** Image obtained from STRING showing upregulated transcripts related to the NF-Kappa B and TNF signalling pathways in *P. multocida* infected MTECs. **G**, **H** Gene ontology analyses of upregulated (**G**) and downregulated (**H**) transcripts in *P. multocida* infected MTECs. Scale bar in B represents 5 μm. Scale bar in C and D represents 20 μm.

At this point and based on our previous studies with epithelial cells, we hypothesized that a *Pasteurella* infection model system could be used to search for new products to combat or prevent opportunistic coinfections. We started by characterizing a *Pasteurella multocida* infection in mouse tracheal epithelial cells (MTECs). By using the same approach developed by Garrido-Jimenez et al. in 2021 [23], we exposed fully differentiated airway epithelial cells to *P. multocida* for 4 h and allowed the epithelial response for 12 additional hours (Figure 3A). After 16 h, we did not find any significant difference in the epithelial barrier function of the epithelia, measured by TEER (Additional file 2). Furthermore, we did not detect morphological changes of the epithelial (Additional file 2). To further confirm the lack of major morphological changes, we performed immunofluorescence against acetylated tubulin to observe cilia in multiciliated cells (MCC). In addition, we also stained with phalloidin to image the actin cytoskeleton and DAPI to observe nuclei. After confocal imaging, we found that all those structures, cilia, actin cytoskeleton and nuclei, were very much comparable to control conditions in *P. multocida* infected epithelia (Figure 3C). A close observation using scanning electron microscopy revealed small colonies of *P. multocida* in top of the epithelium but no morphological changes in adjacent epithelial cells (Figure 3B).

To continue studying the response to *P. multocida* infection, we performed transcriptomic analyses from control and *P. multocida* infected airway epithelial cells. As shown in Figure 3, *Pasteurella* infection increased the expression of 618 transcripts and decreased the expression of 317 transcripts (Figure 3E) in MTECs. Out of the 618 significantly overexpressed transcripts, in the top 10 overexpressed genes, we found C-X-C Motif Chemokine Ligand 1 and 5 (Cxcl1 and Cxcl5), the C–C Motif Chemokine Ligand 20 (Ccl20), the colony stimulating factor 3 (Csf3, also known as G-CSF) and TNF Alpha Induced Protein 2 (Tnfaip2). In addition, two signalling pathways were also significantly represented in the overexpressed gene list, the NF-kappa B and TNF signalling pathways (Figure 3F). Gene ontology analyses revealed that transcripts from the immune response were significantly represented (Figure 3G). On the other hand, out the 317 downregulated transcripts, we found genes involved in viral response and defence (Figure 3H).

Overall, we discovered a scenario where *P. multocida* infection initiated a transcriptional program leading to airway epithelial cells response, without any apparent morphological changes.

### Inactivated LS (i-LS) triggers the innate immune response

So far, we have found that i-LS do not affect the monolayer integrity, and hence its epithelial barrier function. But, does i-LS provoke any effect on the epithelial monolayer? And if yes, could that be positive to treat *P. multocida* infected airway epithelia? To explore those two questions, we decided to characterize and compare the transcriptional program of i-LS treated cells in the presence of absence of *P. multocida*.

First, we checked the transcriptional profile of i-LS treated cells compared to control conditions. We found that only a few transcripts were significantly overexpressed and transcripts were downregulated (Additional file 3). Importantly, the GO analyses revealed that that i-LS treatment triggers the immune system process and the response to virus pathway (Additional file 3). So far, we have found that the i-LS treatment does not affect the airway epithelia in terms of proliferation, barrier function or morphology, but causes an immune response which could be useful to prevent or treat infections.

**Figure 4:**
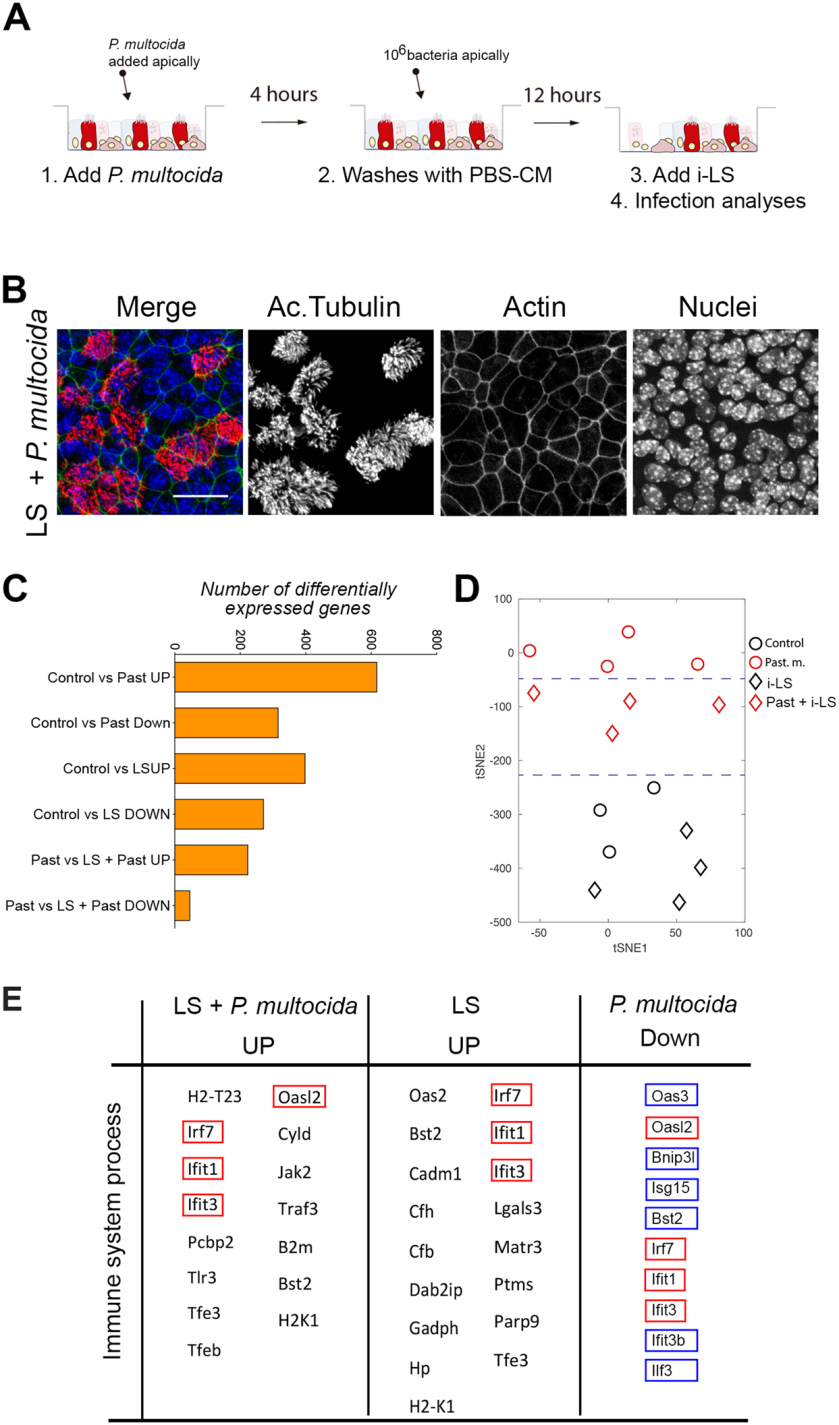
**Inactivated BAL 5 treatment reverts the transcriptional program induced by Pasteurella multocida infection in the airway epithelia. A** Schematic representation of the airway epithelial monolayers infection, treatment and processing protocol. **B** Maximal projection of confocal images for acetylated tubulin (in red), phalloidin (in green) and nucleus (in blue) in airway epithelia infected with *P. multocida* and treated with i-LS. **C** Number of differentially expressed transcripts in different experimental conditions and comparisons. (**D)** tSNE result of the analyses of the 15 RNA-seq samples from different conditions. **E** Table including transcripts related with immune system in different experimental conditions. Red box indicates upregulated transcripts in cells treated with i-LS and infected with *P. multocida*, which were downregulated in *P. multocida* infected cells. Blue box indicates downregulated transcripts in *P. multocida* infected cells, which were not differentially expressed in cells treated with i-LS treated and infected with *P. multocida*. Scale bar in B represents 20 μm.

Based on the above, we set up a protocol to infect and treat airway epithelial cells (Figure 4A). We checked that, as expected, no morphological changes were apparent after 12 h of treatment (Figure 4B). Finally, we performed transcriptomic analyses to compare the molecular response to *P. multocida*, i-LS and the combinations of both. In these analyses, we found that inactivated LS treatment partially reverts the transcriptional program induced by *Pasteurella multocida* infection (Figures 4C–E).

## Discussion

The search for new infectious diseases therapies has increased during the past few years mainly due to two global health issues. On one side, the increase in antimicrobial resistance compromising bacterial diseases treatment and, on the other side, the emergence and re-emergence of viral diseases together with the lack of efficacious antiviral therapies. Therapies targeting the immune system are widespread in human medicine, especially in case of cancer, autoimmune disorders, or in organ transplantation. However, less is known about their current use in infectious diseases control. Microbiota of humans and animals perform fundamental functions in the defence against pathogenic agents and could be employed as a natural source for new immunomodulatory products. Thus, the development of physiologically relevant model systems and protocols to test those natural products is a challenge. We designed and implemented a respiratory infection model and tested different bacteria-derived components with the ability to boost innate immune responses against the pathogen *Pasteurella multocida*. Three products were obtained by the registered strain *Lactobacillus salivarius* CECT 9609, a bacterial specie widely used in commercial products: cell-free supernatants (SN), alive microbial cells (A-LS) and heat-inactivated microbial cells (i-LS).

Out of our three products, only SN and high concentrations of A-LS resulted in a detrimental effect on BSCs proliferation, most probably due to the presence of toxic by-products including organic acids. While A-LS altered the morphology and thus caused a decrease on the epithelial barrier, i-LS did not affect the barrier function of the airway epithelia. Besides, i-LS triggered important immune system processes, including anti-viral responses, as was hitherto reported in *L. salivarius* previous studies [24]. Actually, immune response boosted by LAB has led to their potential usage as mucosal-delivery vehicles [25, 26].

*Pasteurella multocida* is a common respiratory pathogen leading to important economic losses in livestock. In contrast, respiratory infections in humans are relatively uncommon and may occur from a zoonotic transmission. *Pasteurella* species can be considered as potentially severe coinfection agents with either viral or bacterial infections, especially in immunocompromised patients [11, 13]. A 4 h-exposition to microbial cells followed by a longer period of response did not affect any of the epithelial structures or the epithelial barrier function, in contrast to previous studies with similar approaches [27, 28]. However, that exposition was enough to trigger a transcriptional program leading to the epithelial cells response. The overexpression of genes involved in the immune response, including NF-KB and TNF signalling pathways and chemokine mediators, seems to be relevant for inflammatory response against *Pasteurella* species, as was previously described in different cell types [27, 29]. Furthermore, genes related to antiviral immune response and defence were downregulated in airway epithelium exposed to microbial cells. Thus, *P. multocida* could exacerbate an existing viral infection or may benefit from a viral infection, which highlights its role as opportunistic pathogen.

Altogether, when we combined *P. multocida* infection model and i-LS, we found that exposure to heat-inactivated microbial cells reverted the transcriptional program induced by the pathogen. Modulation of exacerbated proinflammatory effects may favour the outcome of the disease, diminishing tissue damage and severe complications. While interferons and interferon-inducible proteins like IRF7, IFIT1, and IFIT3 are primarily associated with antiviral defence [30], they can also be induced in response to bacterial ligands, contributing to the resolution of the infection [31, 32]. However, the specific contribution of these factors in combating *Pasteurella* infections is not fully understood. In addition, key genes related to anti-viral response that were down regulated in the presence of the pathogen, seemed to be up-regulated when i-LS is added to the infection model. In a multi-microbial scenario, it is generally assumed that primary infection occurs from viral agents that prejudice the host to opportunistic bacterial infections [33]. However, our results highlight the role of commensal bacteria with the ability to downregulate viral immune responses and therefore rendering the host less susceptible to secondary viral infections. It is well known the ability of pathogenic bacteria to depress viral response [33], but little is known about the role of beneficial bacteria within this scenario.

The in vitro experimental model we assessed serves as a basis for the study of the protective function of beneficial bacteria derivatives in the outcome of a respiratory bacterial infection. Our experimental design meets our criteria for cellular model system replication and validates our bacteria-derived components selection. It constitutes the first step to detect candidates to elaborate a product aimed at combating infectious diseases before any in vivo evaluation. Our studies have increased the available knowledge on the interaction between the host and its microbiota and show great prospects for the therapeutical use of innovative elements to control respiratory tract infections.

### Supplementary Information

**Additional file 1. A-LS treatment produce morphological changes in airway epithelial cells.**

**Additional file 2. Pasteurella infection at early stages do not affect the airway epithelium.**

**Additional file 3. Transcriptional response to i-LS and** ***Pasteurella multocida***.

## Data Availability

Datasets generated during the current study are available from the corresponding author on reasonable request.
