# Supplementary figures and images for: Immunomodulatory effects of inactivated Ligilactobacillus salivarius CECT 9609 on respiratory epithelial cells
Source: Vet Res. 2023 Oct 16;54:91. doi: 10.1186/s13567-023-01228-z (PMC10580541; doi:10.1186/s13567-023-01228-z)

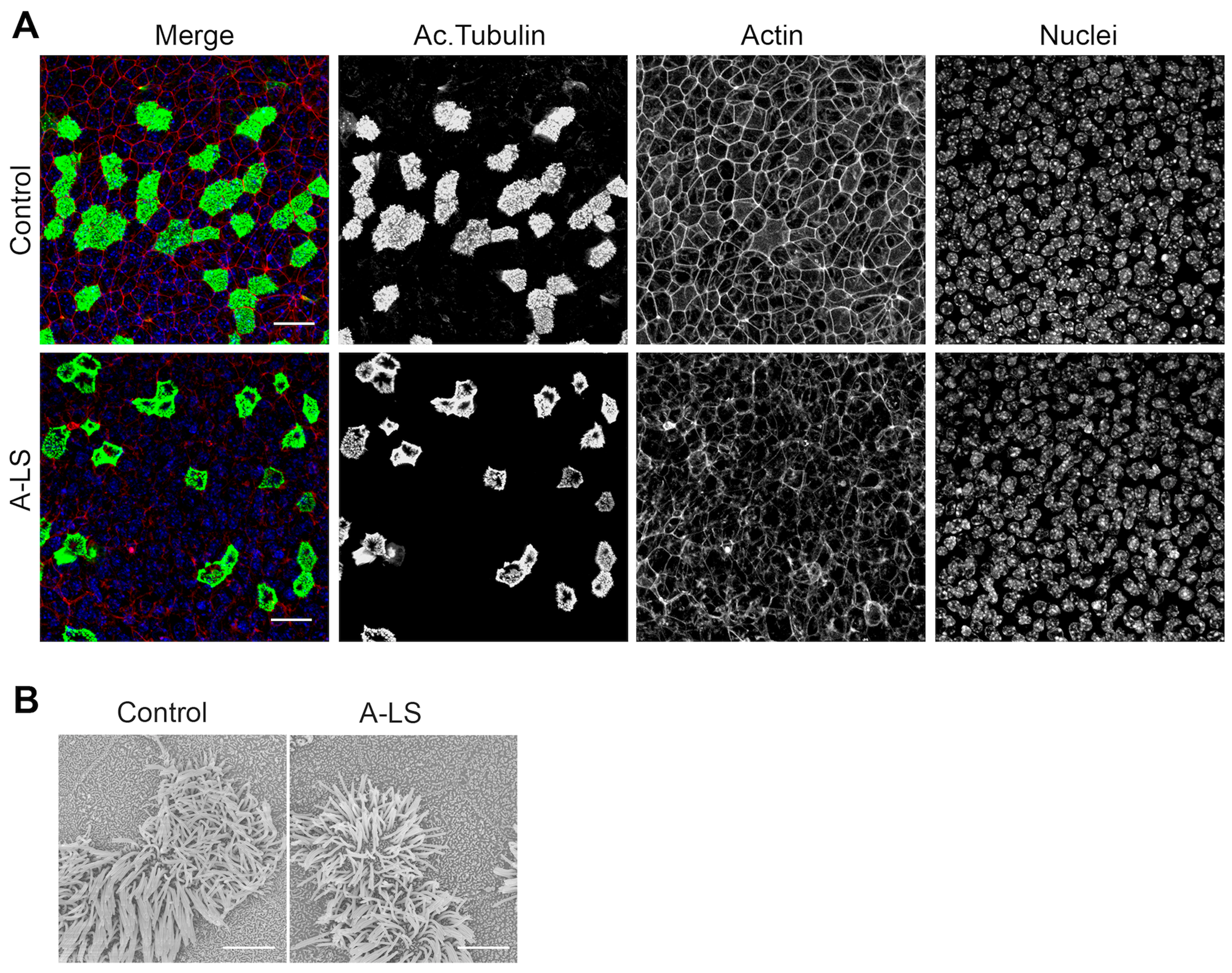

Supplement: Supplementary file 1 — Additional file 1. A-LS treatment produce morphological changes in airway epithelial cells. [file 13567_2023_1228_MOESM1_ESM.tif]

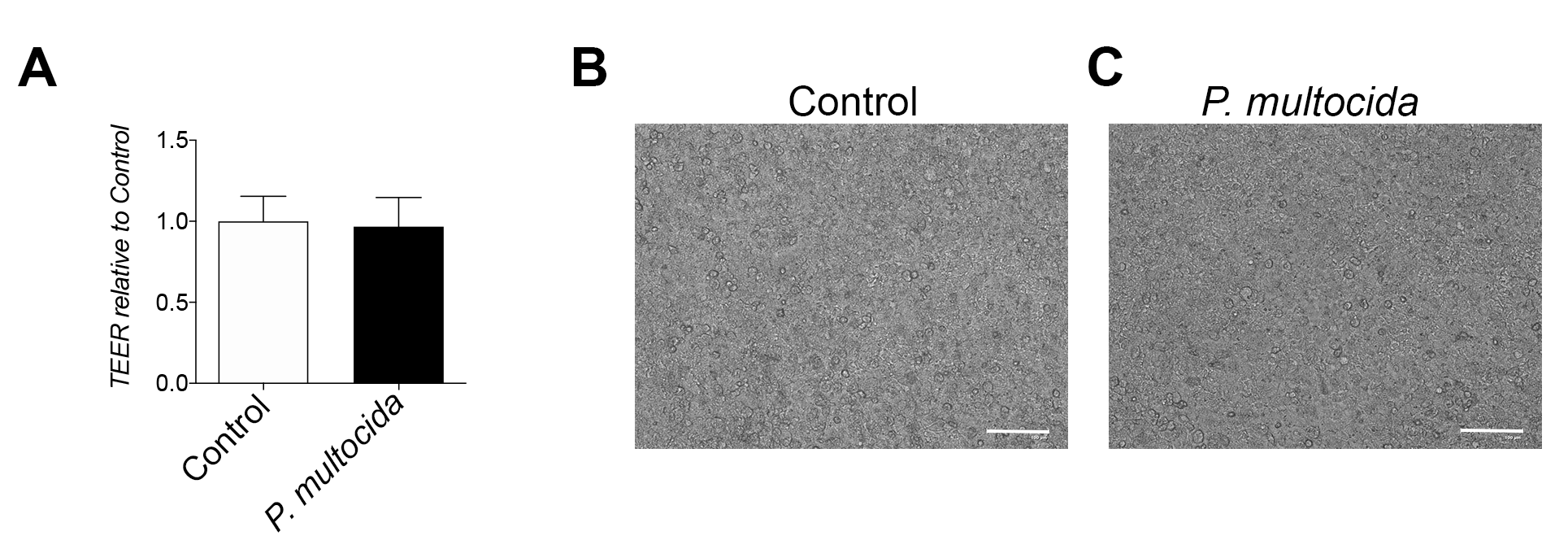

Supplement: Supplementary file 2 — Additional file 2. Pasteurella infection at early stages do not affect the airway epithelium. [file 13567_2023_1228_MOESM2_ESM.tif]

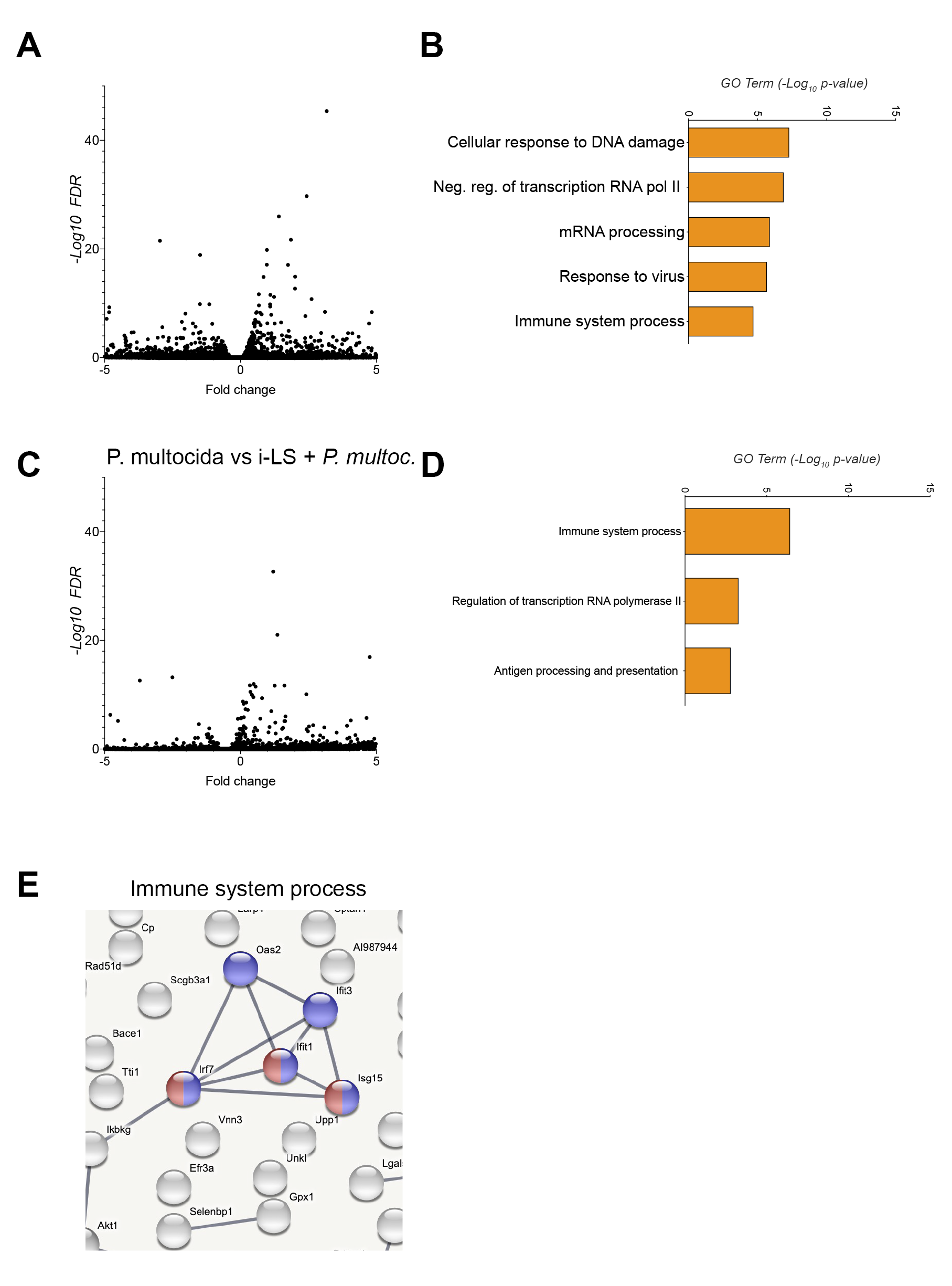

Supplement: Supplementary file 3 — Additional file 3. Transcriptional response to i-LS and Pasteurella multocida. [file 13567_2023_1228_MOESM3_ESM.tif]
